# Holographic diffusers for endoluminal-scale optical ultrasound imaging

**DOI:** 10.1117/1.JBO.31.1.016006

**Published:** 2026-01-23

**Authors:** Fraser T. Watt, Efthymios Maneas, Desta Chan, Edward Z. Zhang, Paul C. Beard, Erwin J. Alles

**Affiliations:** a University College London, UCL Hawkes Institute, Faculty of Engineering Sciences, London, United Kingdom; b University College London, Department of Medical Physics & Biomedical Engineering, Faculty of Engineering Sciences, London, United Kingdom

**Keywords:** holographic diffuser, holographic optical element, optical ultrasound, real-time imaging, endoluminal imaging

## Abstract

**Significance:**

Freehand optical ultrasound (OpUS) is an emerging imaging modality that utilizes arrays of fiber-optic, photoacoustical ultrasound sources, and a single fiber-optic detector to perform pulse-echo ultrasound imaging in real time and at video rate. The freehand OpUS imaging probes presented to date have utilized either flat-cleaved optical fibers to generate an array of small, circular sources, or featured an additional array of eccentric optical waveguides to generate elliptical sources that improve ultrasound directivity. However, the incorporation of waveguides imposed severe restrictions on the size of the sources, the source pitch within the array, and the overall size of the imaging probe, and ultimately limited achievable image quality and clinical utility of freehand OpUS probes.

**Aim:**

We present an alternative method for generating eccentric fiber-optic OpUS sources, which incorporates an anisotropic holographic diffuser element (HDE) to shape the profile of fiber-delivered excitation light.

**Approach:**

A commercially available HDE was selected and imprinted into a UV-curable adhesive to improve optical resilience. The monolithic and near-uniform structure of the resulting adhesive-imprinted HDE structure greatly facilitated alignment with, and allowed for arbitrary positioning of, arrays of optical fibers, without adding significant bulk.

**Results:**

The use of an HDE enabled dense, cladding-to-cladding packing of optical fibers, resulting in the first freehand OpUS probe featuring an acoustic source pitch below the spatial Nyquist limit (thus eliminating grating lobe artifacts), high channel count (144 sources), and endoluminal-scale physical dimensions (diameter: 18.5 mm).

**Conclusion:**

This HDE-enabled freehand OpUS imaging probe achieved video-rate, real-time imaging at increased image quality and depth and allowed for the first endoluminal freehand OpUS imaging within a realistic imaging phantom.

## 1 Introduction

Optical ultrasound (OpUS) imaging probes generate and detect ultrasound through optical means. An optically absorbing material is illuminated by a pulsed light source, thus generating broadband ultrasound via the photoacoustic effect^1^^,^^2^ that is transmitted into the human body and backscatters off tissue interfaces. Detection of the back-scattered (“pulse-echo”) ultrasound signals is performed optically by monitoring a highly sensitive optically resonant structure such as fiber-mounted Fabry–Pérot cavities^3^^,^^4^ or ring resonators,^5^ or through free-space optical interferometry.^6^^,^^7^ Different types of OpUS imaging systems have been presented: free-space systems have achieved pulse-echo imaging in water baths,^6^^,^^8^^,^^9^ or optical ultrasound excitation and interrogation have been achieved through air to enable noncontact OpUS imaging.^7^^,^^10^ In addition, OpUS imaging on an interventional scale has been presented, where miniature fiber-optic probes were translated across samples and phantoms to synthesize imaging apertures.^11^^–^^15^ Recently, such a miniature fiber-optic probe achieved the first real-time and video-rate (up to 7 Hz) interventional-scale OpUS imaging.^16^ However, free-space systems require the imaging target to be either fully submerged or stationary, which is unsuitable for clinical settings. Mechanical scanning of interventional probes is either too slow to achieve real-time imaging or limited to a side-viewing orientation and is not practical due to the size constraints associated with endoluminal scenarios.

By contrast, freehand OpUS probes combine an array of sequentially illuminated fiber-optic OpUS sources with a single fiber-optic detector to enable rapid synthesis of an imaging aperture using fast steerable optics. Such systems can achieve real-time imaging at a frame rate of up to 24 Hz,^17^ using a probe form factor that approximates the size and shape of a conventional electronic handheld ultrasound probe^17^^,^^18^ and allows for flexible, handheld operation. Such systems have achieved resolutions down to 125  μm (lateral) and 173  μm (axial) at imaging depths of ca. 20 mm, and the lack of electronic or mechanical components at the distal end renders freehand OpUS probes inherently compatible with MRI and CT imaging systems.^19^ Although currently a low channel count (64) limits the image contrast of freehand OpUS imaging probes, their clinical relevance has already been demonstrated through *in vivo* imaging.^17^^,^^18^

Fiber-optic OpUS sources typically exhibit circular symmetry and hence result in small, circular OpUS sources. However, OpUS sources ideally exhibit eccentric geometries:^9^ narrow in the lateral (in-plane) direction to produce a highly divergent acoustic field, yet wide in the elevational (out-of-plane) direction, which effectively collimates the acoustic field to limit geometrical attenuation and avoids image clutter generated by out-of-plane contrast. A previous freehand OpUS probe incorporated a 3D-printed array of eccentric optical waveguides^17^ to effectively shape the excitation light. However, the 3D printer employed achieved an element pitch (400  μm) exceeding the spatial Nyquist criterion, resulting in significant grating lobe artifacts. Although these artifacts could be reduced through signal processing,^20^^,^^21^ greater numbers of more densely packed sources would be required for full suppression. However, alternative light-shaping waveguide fabrication methods (e.g., high-resolution 3D printing or selective laser etching, microlens arrays, eccentric capillaries, compressed polymer optical fibers, and noncircular optical fiber)^22^ suffer from significant cost, poor reproducibility, limited versatility and availability, and added bulk. In addition, any method involving arrays of discrete beam shaping elements necessitates accurate, simultaneous alignment of the array with dozens to hundreds of optical fibers, which requires specialist assembly and equipment.

One synergistic solution to shaping the excitation light delivered through dense fiber arrays can be found in holographic optical elements (HOEs), which comprise thin, holographic structures that can achieve a wide range of functions. For instance, HOEs have been presented that achieve collimation, focusing, and coupling.^23^^–^^26^ Of particular interest are holographic diffuser elements (HDEs), which are commonly applied to homogenize room lighting and are commercially available in both isotropic and anisotropic versions. HDEs are typically monolithic films of near-uniform structure, which relaxes tight alignment criteria and allows for arbitrarily dense fiber placement.

Here, a next-generation freehand OpUS probe is presented that incorporates an anisotropic HDE, which allows for dense (exceeding cladding-to-cladding) fiber positioning while simplifying fabrication and avoiding the additional bulk typically associated with waveguide or microlens arrays. This approach allows for sub-Nyquist source spacing and substantially higher channel counts, which substantially improves the imaging performance, yet results in a significantly smaller, endoluminal-scale imaging probe. This paper presents the probe fabrication, optical and acoustical characterization, imaging performance, and the first endoluminal application—including an assessment of its compatibility with CT imaging—of a next-generation endoluminal-scale freehand OpUS imaging probe.

## 2 Methods

### 2.1 Holographic Diffuser Element

HDEs are commercially available in large, cost-effective monolithic sheets that feature a grating-style holographic structure fabricated on the surface of a substrate. The substrate is typically a semirigid material to improve mechanical stability. Such HDEs offer versatility in diffuser angle (ranging between ca. 1 and 80 deg) and can achieve selective, anisotropic diffusion. In this work, an HDE designed to achieve a diffusion angle of 60 deg along one direction and 1 deg in the orthogonal direction is considered (L60X1E5-12 High Tech P1, Luminit, Torrance, California, United States), which is fabricated onto a polycarbonate substrate and exhibits a transmissivity of 89% across the visible and near infrared spectra.

**Fig. 1:**
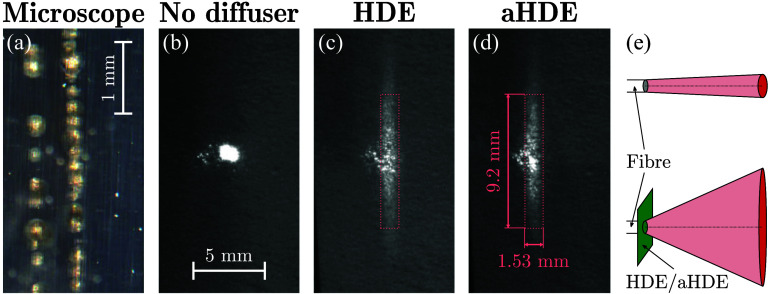
Comparison of holographic diffuser elements (HDEs). (a) Microscope image of the structured side of a commercial HDE after fiber-delivered illumination through the substrate. Burn-in damage (yellowed areas) is observed within the HDE substrate in the majority of illuminated areas within seconds of exposure. Note that the holographic structure appears undamaged. (b)–(d) Images of the light distribution resulting from propagation through (b) no diffuser, (c) a commercial HDE, and (d) an adhesive-imprint HDE (aHDE). Beam profiles measured at a distance of 20 mm behind the shaping elements. Panels (b)–(d) are shown on the same scale. (e) Schematic of the symmetric illumination pattern generated by a fiber in the absence of a diffuser (top) and the eccentric pattern in the presence of an HDE or aHDE (bottom).

Although such HDEs are highly durable at the low intensities typical for room lighting applications, OpUS sources are typically excited using nanosecond pulsed laser light (wavelength: 1064 nm, pulse energy: several tens of μJ, pulse duration: 1 to 2 ns) fiber-delivered to a very small area (core diameter: 105  μm), resulting in a high intensity of ca. $200\;\mathrm{MW}/{\mathrm{cm}}^{2}$ that results in damage to the HDE substrate [Fig. 1(a)]. Therefore, instead of using the HDE as supplied, its negative imprint was created by mold-casting the holographic surface structure using a UV-curable adhesive (NOA81, Norland Products, Monroe Township, New Jersey, United States) with a refractive index [1.56 (Ref. 27)] closely matching that of the HDE [1.58 (Ref. 28)]. This adhesive, which had previously been observed to withstand the intensities typical for OpUS imaging, was deposited onto the HDE surface, pressed flat with a microscope slide, and cured. Once cured, the adhesive was readily peeled from both the microscope slide and the polycarbonate HDE, resulting in a negative imprint of the holographic structure. Tests performed using collimated visible light (wavelength: 635 nm; PL202, Thorlabs, Newton, New Jersey, United States) confirmed that such an adhesive-HDE (aHDE) achieved similar eccentric optical diffusion [Figs. 1(b)–(1d)], despite a different refractive index and inverted structure. The aHDE fabrication process is shown step-by-step in Fig. S1 in the Supplementary Material.

### 2.2 OpUS Probe Assembly

The aHDEs considered here are monolithic and near-uniform in structure, which greatly facilitates their incorporation into OpUS imaging probes comprising hundreds of fiber-optic sources. The corresponding relaxed alignment requirements enable manual and in-house assembly, which in turn allows for rapid and cost-effective iterative prototyping. This aHDE uniformity is exploited here to achieve an OpUS source density limited only by the physical dimensions of the optical fibers, and optical shaping is achieved by simply placing an aHDE across the custom fiber bundle front face.

#### 2.2.1 Source array fabrication

**Fig. 2:**
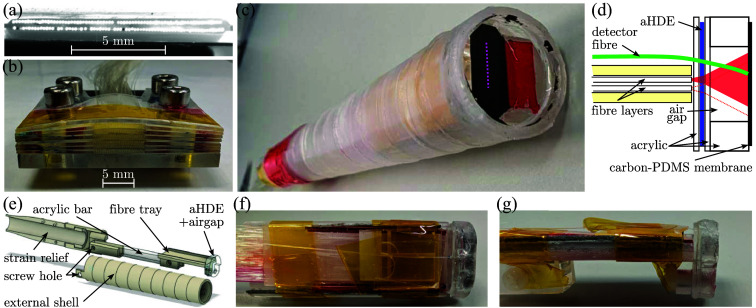
Endoluminal-scale OpUS probe assembly. (a)–(b) The proximal (a) and distal (b) ends of the custom array comprising 144 fibers. Distally, the fibers are arranged in two sheets of 72 fibers arranged cladding-to-cladding; proximally, the fibers are distributed across six sets to minimize optical cross-talk. The fibers in the distal image (a) appearing dark or missing are a result of imprecise alignment of the through-illumination light source—all 144 fibers remained effective optical waveguides. (c) Photograph of the assembled endoluminal-scale OpUS imaging probe measuring 18.5 mm in diameter. The imaging aperture is indicated by the dotted magenta line. Sections of red acrylic tape (thickness: 25  μm) are visible, which are used to accurately adjust spacings between the various probe elements. (d) Schematic of the assembly used to shape and deliver the excitation light (indicated in red). An adhesive-imprint holographic diffusion element (aHDE) is sandwiched between two acrylic sheets, and a third acrylic component provides a 3-mm-thick air gap to allow the light to spread eccentrically before being photoacoustically converted into ultrasound within a carbon-loaded PDMS membrane stretched across the air gap. A single fiber-optic ultrasound detector running above the fiber array and through the air gap is positioned centrally within the OpUS source array and placed in contact with the carbon-PDMS membrane. (e) CAD rendering of the 3D printed assembly. A custom tray held the fiber array, aHDE, and air gap components. A rigid acrylic bar firmly mounted these components to an elastic strain relief and a single nylon screw joined all pieces. Photographs of the assembled custom fiber tray in top-down (f) and side profile (g).

To achieve dense fiber-optic source packing, 1.5 m long sections of multimode optical fiber (core diameter: 105  μm; cladding diameter: 125  μm; numerical aperture: 0.22; FG105LCA, Thorlabs) were aligned cladding-to-cladding and bonded to acrylic substrates. Two such sheets of fiber were fabricated, and one sheet was inverted and laterally offset by half of the fiber pitch before affixing, thus resulting in a two-layer array of fibers with a lateral fiber pitch of 62.5  μm [Fig. 2(a)]. This array would hence satisfy the spatial Nyquist criterion for acoustic frequencies up to 12 MHz, which covers a substantial range of the acoustic bandwidth typically generated in OpUS systems.^2^

Each sheet of 72 fibers was fabricated by manually stripping each fiber section down to the cladding. A custom acrylic substrate (thickness: 0.5 mm) was prepared to include an engraved strip (width: 9.5 mm; cut and engraved using commercial laser cutter VLS 4.60, Universal Laser Systems, Scottsdale, Arizona, United States). The 72 fibers were coarsely aligned and set on the substrate, pressure was applied onto the fibers via a microscope slide clamped in place above the fiber array, and the substrate was translated orthogonal to the fibers’ long axes, which allowed for the fibers to roll into the strip and self-align into a cladding-to-cladding configuration. The fibers were then bonded to the acrylic substrate using the NOA81 UV-curable adhesive.

The two sheets were then inverted, aligned using a manual micropositioner to achieve a half-pitch lateral offset, and bonded using NOA81 adhesive [Fig. 2(d)]. Finally, the resulting acrylic–fibers–acrylic sandwich was laser cleaved and manually polished using lapping sheets to ensure a flat, smooth, and optically clear distal surface. Note that due to excess adhesive, the two sheets of fiber were separated by ca. 300  μm; however, the OpUS probe is designed to comprise eccentric sources measuring several millimeters in the elevational direction, and this sheet separation is hence negligible. At the proximal end, the 144 fiber sections were again manually stripped, aligned individually along 24 grooves (spaced 400  μm apart) engraved into six separate acrylic substrates, and affixed such that the fiber tips protruded from the substrate edge [Fig. 2(b)]. After laser cleaving and polishing, these six fiber arrays were sandwiched and positioned in the focal plane of a scan lens so that excitation light could exclusively be coupled into the core of each fiber, thus avoiding damage from exposing coatings, adhesives, or substrates to high-intensity light.

#### 2.2.2 Light-shaping element

The mold-cast thin and flexible aHDE was sandwiched between two acrylic substrates (thickness: 0.5 mm) to improve rigidity and scratch resistance. In addition, the aHDE was sealed along all edges to the substrates using NOA81 adhesive to prevent water ingress in case the OpUS probe is fully submerged; surface HDEs are typically designed to operate in air, and water ingress would deteriorate its performance.

To use the aHDE for OpUS generation, transmitted light required a propagation distance to expand to the desired eccentric shape. A previously reported proof-of-concept OpUS source used a solid backing of clear acrylic to allow the light to expand before illuminating an OpUS-generating membrane.^22^ However, initial tests using this configuration displayed strong acoustic ringing within the acrylic. To avoid the associated imaging artifacts, here, an air-backed transducer design was deployed. A 3-mm-thick acrylic piece with a rectangular hole was fixed with adhesive to a section of the aHDE-acrylic sandwich, where the hole acted as a stand-off to allow the excitation light to eccentrically expand.

An elemental-carbon-doped polydimethylsiloxane (PDMS) membrane (thickness: 25  μm^7^) was stretched over the stand-off hole, and the edges of the membrane were sealed with adhesive to prevent water ingress from wet environments. The acoustic impedance mismatch between the membrane and the air ensured ultrasound was predominantly transmitted away from the air gap, thus eliminating acoustic ringing within the probe. A schematic of this assembly is shown in Fig. 2(d).

#### 2.2.3 Fiber-optic ultrasound detector

Pulse-echo signals were received using a single fiber-optic Fabry–Pérot ultrasound detector.^3^^,^^4^ Unlike in previously presented systems,^17^^,^^18^ where the substrates and light shaping components were solid and the detector had to be placed outside of these components or even protrude beyond the probe front surface, the air gapped approach used here provides an opportunity to place this fragile detector fiber inside the OpUS imaging probe. This approach simultaneously allows for the use of a monolithic membrane without perforations, protects the sensor, and minimizes the probe footprint.

The detector fiber was run over the top of the fiber array and through a notch created in the aHDE substrate. This notch pushed the fiber down such that the detector at the tip was positioned centrally within the OpUS source array. Using a custom laser-cut acrylic “fiber launch,” the detector fiber was bent to ensure the tip was located within the center of the eccentric OpUS sources—and thus within the OpUS image plane—and pushed gently against the stretched PDMS membrane to achieve acoustic coupling while avoiding buckling of the membrane [Figs. 2(f)–2(g)]. Although the PDMS membrane and mechanical coupling (rather than conventional “wet” coupling using, e.g., coupling gel) introduce acoustical attenuation,^29^ and the presence of the fiber within the air gap might result in mild shadowing or scattering of the excitation light, this approach was preferred as positioning the detector within the image plane maximizes signal strength and allows for an intact PDMS membrane to ensure a watertight air gap.

#### 2.2.4 Endoluminal-scale housing

The components described above were fixed to a custom frame and enclosed in a protective shell [Figs. 2(c) and 2(e)]. Both the frame and shell were printed in a clear resin (RS-F2-GPCL-04, Clear Resin V4, Formlabs, Somerville, Massachusetts, United States) using a commercially available resin 3D printer (Form 3B, Formlabs; layer height: 25  μm). The protective shell measured 98 mm in length and was constrained to an outer diameter of 18.5 mm (for an imaging aperture width of 9.5 mm), which falls well within the upper limit of 24 mm for endoluminal probes that can be applied without patient discomfort.^30^ The protective shell was designed to exclusively comprise plastics, glass, and adhesives to ensure insensitivity to electromagnetic interference and required just a single nylon screw to allow for easy disassembly to facilitate modifications and repairs. Finally, the protective shell comprises a protruding ring [Fig. 2(c)] to prevent deformation of the ultrasound generating membrane due to physical contact with the phantoms.

### 2.3 OpUS Image Acquisition

The proximal end of the endoluminal-scale OpUS probe was connected to a previously reported freehand OpUS acquisition console.^17^ Using this console, pulsed excitation light (wavelength: 1064 nm; pulse duration: 1.5 ns; pulse repetition rate: 2.7 kHz; maximum pulse energy: 71  μJ; DSS1064-Q3, Crylas, Berlin, Germany) was sequentially coupled into the fibers in the probe bundle by steering the beam with scanned galvanometer (galvo) mirrors (GVS002, Thorlabs). The beam was focused onto the fiber tips by a scan lens (focal length: 110 mm; field of view: 28.9  mm×28.9  mm; LSM05-BB, Thorlabs, Germany) and traversed a raster scan pattern to illuminate each row of 24 fibers in the proximal end of the probe bundle in a stop–start fashion. The excitation light was converted into ultrasound within the carbon-PDMS membrane, where each of the 144 fibers sequentially generated an eccentric optical ultrasound source.

Back-scattered ultrasound modulated the thickness of the cavity in the Fabry–Pérot detector, which was interrogated by a tunable continuous-wave light source (wavelength range: 1500 to 1630 nm; TUNICS T100S-HP, EXFO, Canada) via a circulator (6015-3-APC, Thorlabs), and tuned to the maximum sensitivity of the cavity in the detector.^3^ The DC component of the photodiode signal (0 to 500 kHz) was used to maintain the sensor sensitivity; the high-frequency component (>500  kHz) contained the pulse-echo ultrasound signal. Each fiber-optic source generated a single pulse-echo signal (an “A-line”); the resulting 144 A-lines were gathered in a B-scan for signal processing and image reconstruction.

The pulse-echo B-scan was sampled using a high-speed digitizer (sampling rate: 125 MHz; resolution: 16 bit; M4i.4420-x8, Spectrum, Germany) installed in a rack-mounted blade-PC (Precision 3930 Rack, Dell Corporation, Round Rock, Texas, United States; CPU: Intel Core i7-8700K, RAM: 128 GB). Each B-scan was bandpass filtered (range = 2 to 15 MHz) and passed through a GPU-enabled beamformer for video-rate image reconstruction. The reconstructed image was subsequently envelope-detected and log-compressed and displayed in real time. A parallelized data acquisition approach achieved real-time “delay-and-sum” (DaS) reconstruction equivalent to “dynamic focusing”^31^ at a frame rate of 12.2 and 10.1 Hz for imaging depths of 24 and 43 mm, respectively.^17^

The imaging system was capable of video-rate DaS reconstruction and offered an additional nonlinear reconstruction option based on the pseudo-DMaS (PDMaS) beamformer.^21^ With PDMaS, the DMaS algorithm^32^ is applied to improve image clutter rejection, whereas the particular geometry of the OpUS probe (featuring a single central detector and weakly directional sources) mandated alterations to the algorithm to improve both image quality and computational efficiency. Both beamformers were implemented using the NVIDIA CUDA toolbox (v11.2.152) and capable of real-time, video-rate reconstruction when executed on a Quadro P6000 GPU (NVIDIA, Santa Clara, California, United States). Despite lower resolution and signal-to-noise ratio (SNR), DaS beamforming was performed live during image acquisition as the resulting images are typically easier to interpret and compare with other systems, whereas PDMaS was applied offline.

### 2.4 Performance Characterization

The acoustic performance of the endoluminal-scale OpUS probe was characterized through acoustic field scans performed with a calibrated needle hydrophone (calibrated bandwidth: 1 to 30 MHz; diameter: 200  μm; NH0200-SYSTEM, Precision Acoustics, Dorchester, United Kingdom), mounted on a pair of orthogonally mounted motorized stages (MTS50/M-Z8, Thorlabs). The field scan was carried out over an area measuring 13 mm (wide) by 4 mm (tall), with an isotropic step size of 50  μm, in a plane parallel to the probe face at a distance of 3 mm. At each hydrophone position, all 144 OpUS sources were excited to generate 144 separate field scans. Data were acquired using 30-fold averaging to ensure good SNR. The field scan was performed at 30% laser power (pulse energy: 21.3  μJ) to reduce the risk of optical damage to any optical components over the extended duration of the field scan (ca. 45 h). The field scan data were numerically propagated using the angular spectrum approach^33^ to assess the source shape and directivity at varying distances from the probe. Additional on-axis hydrophone measurements were made at 3 mm from the probe face at the full laser power (pulse energy: 71  μJ) used during OpUS imaging to assess the ultrasound generation at the laser powers used for imaging.

The imaging performance of the probe was characterized using thin tungsten wires placed orthogonal to the imaging plane in a water bath. First, to measure the image resolution at varying axial depth, a single tungsten wire (diameter: 27  μm) was placed centrally within the image, and manually translated via a micropositioner between depths of 3 and 24 mm under continuous OpUS imaging. To improve SNR, 10-fold averaging was used for this phantom only. The lateral and axial resolutions were then extracted at the full-width-at-half-maximum (FWHM) of the images of the wire. A second phantom comprising several layers of thicker tungsten wires (diameter: 75  μm), spaced 2 mm apart, was used to assess the imaging performance for close-packed point-like targets, which proved challenging in previously reported work.^17^

### 2.5 Endoluminal Demonstration

To assess the capability of the endoluminal OpUS probe in more clinically relevant settings, an endobronchial ultrasound (EBUS) tissue-mimicking phantom was imaged. The phantom comprised a block of 10% w/w poly(vinyl) alcohol (PVA) cryogel^34^^,^^35^ with external dimensions of 17 cm (wide) by 17 cm (deep) by 8 cm (tall). The phantom contained a set of wall-less cavities modeled after a section of the trachea and the primary and secondary bronchi. Spherical inclusions exhibiting physiologically accurate acoustic properties were placed in locations coinciding with the lymph nodes. Although the OpUS probe was not intended to replicate the function of a conventional EBUS probe, which are typically side-viewing and flexible, the trachea cavity (diameter: 18 mm) allowed for insertion of the endoluminal OpUS probe and enabled the first OpUS imaging using a freehand OpUS probe in a tissue-mimicking, endoluminal setting.

One of the lymph node inclusions was located directly behind the carina (the apex of the primary branch point) and was the target in this imaging study. The phantom was submerged in deionized water for acoustical coupling. Once fully inserted, the OpUS probe was manually tilted and translated to control the visualization of the internal features of the phantom. Cone-beam CT (CBCT; O-arm Intraoperative Scanner, Medtronic, Ireland) imaging was performed of the phantom (in air) with the OpUS probe inserted—but not actively imaging—to confirm the probe positioning relative to the phantom geometry.

## 3 Results

### 3.1 Acoustical Characterization

**Fig. 3:**
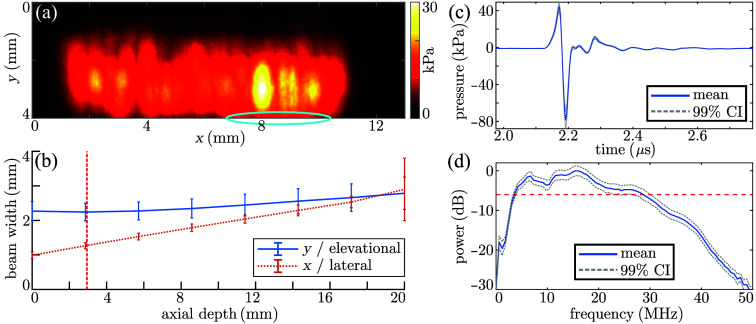
Acoustical characterization of the freehand OpUS probe. (a) Maximum-intensity projection of the 144 compounded back-propagated field scans acquired at 30% laser power. The cyan annotation indicates those sources for which the emitted fields are partially clipped. (b) Elevational and lateral beam width for all sources, numerically propagated to varying depths. The field scan measurement plane is indicated by the red dashed line; error bars represent ±1 standard deviation. The 28 clipped field scans are omitted from the error bars. (c) Mean and 99% confidence interval (CI) for 144 on-axis pressure measurements recorded at the full laser power used during imaging. (d) Mean and 99% CI of the acoustic power spectra for all sources. The red dashed line indicates the −6  dB level used to extract the acoustic bandwidth.

Acoustic field scans demonstrated that the fiber array and aHDE resulted in 144 sources of approximately uniform size and shape. When back-propagated to the OpUS-generating PDMS membrane surface, the sources in the array exhibit an eccentric shape with dimensions 0.9±0.3  mm (wide; mean ± standard deviation) by 2.3±0.3  mm (tall). Given the lateral fiber pitch of 62.5  μm, consecutive sources thus exhibit significant spatial overlap, which is evident in the compound field scan [Fig. 3(a)]. The field scans were numerically propagated to distances between 0 and 20 mm to assess the beam divergence [Fig. 3(b)], where the individual field scans computed at a distance of 0 mm are shown in Fig. S2 in the Supplementary Material. Due to a slight misalignment during the field scan, the pressure fields of 28 of the 144 sources were clipped; therefore, to avoid the associated numerical inaccuracies, those 28 sources were excluded in this analysis. The remaining sources exhibited nearly constant elevational beam widths and corresponding image slice thickness of ∼2.3  mm for axial depths of up to 20 mm, which is significantly larger than the out-of-plane offset (ca. 300  μm) between the fiber sheets, and the array can hence be approximated as linear. As intended, the sources rapidly diverge laterally over the imaging plane [Fig. 3(b)].

When measured at full laser power and on-axis (only) at a distance of 3 mm from the PDMS membrane [Fig. 3(c)], the 144 sources exhibited highly uniform performance, with an average peak–peak pressure of 0.12±0.06  MPa. Variations between sources in the recorded pressures can be attributed to changes in ambient conditions and laser power variation over the extended time required to acquire the field scan. This peak–peak pressure was approximately half of that generated using the same light source by a previous freehand probe^17^; however, as the surface area of the OpUS sources generated in the endoluminal-scale was ca. 10-fold greater than in previous work, the aHDE-air gap design appears to transmit the excitation light highly efficiently. The 144 sources exhibited a highly uniform, broad bandwidth of 25±1  MHz evaluated at the −6  dB level [Fig. 3(d)] typical for fiber-optic OpUS sources.^2^ Power levels were normalized to the peak of the average spectrum.

### 3.2 Imaging Performance

**Fig. 4:**
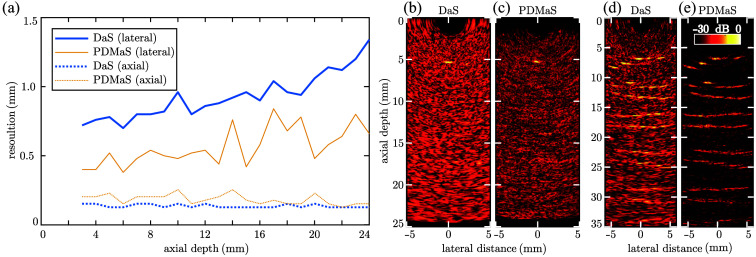
Imaging resolution measurements. (a) Lateral and axial resolutions for the endoluminal-scale OpUS probe, extracted as full-width-at-half-maximum at varying depth from images reconstructed with both delay-and-sum (DaS) and pseudo-DMaS (PDMaS) beamformers. (b), (c) DaS (b) and PDMaS (c) reconstructed images of a single point target centrally located at an axial depth of 5 mm. DaS (d) and PDMaS (e) reconstructed images of multiple point targets positioned at depths of up to 35 mm. All reconstructed images are presented on the same 30 dB dynamic range.

Images of a point scatterer reconstructed using both DaS and psuedo-DMaS exhibited depth-independent axial resolution (ca. 125  μm for DaS and 230  μm for PDMaS) but a lateral resolution that decreased with increasing imaging depth [640 to 1300  μm for DaS, 420 to 800  μm for PDMaS; Fig. 4(a)]. The fluctuations in the lateral resolution observed for PDMaS are due to noise; the PDMaS image amplitude decreases faster than that for DaS [cf. Fig. 4(d)–4e)], and the stochastic nature of noise therefore affects PDMaS images more strongly. The observed lower lateral resolution is a result of the limited aperture width of just 9.5 mm necessitated by the endoluminal scale as the lateral imaging resolution is inversely proportional to the aperture width.^36^ The images obtained with the point scatterer located at a depth of 5 mm [Figs. 4(b)–4(c)] exhibited a contrast of 20 dB (DaS) or 23 dB (PDMaS), which was comparable to that obtained using a previously presented probe.^17^ However, in that work, this contrast was achieved using threefold averaging and a thicker—and hence more echogenic—imaging target, and the resulting image contained significant artifacts due to strong grating lobes. Achieving the same contrast here, without averaging, hence represents a significant increase in imaging performance, which can be attributed to the denser source array and greater ultrasound generation efficiency due to the air-backed film. In addition, the images obtained with the endoluminal-scale OpUS probe are virtually free from grating lobe artifacts. This is further confirmed when imaging a phantom comprising multiple, closely positioned point targets [Figs. 4(d)–4(e)], where the individual targets (diameter: 75  μm) can be resolved up to a depth of 15 mm. This substantial increase in image quality was additionally predicted in the simulations shown in Fig. S3 in the Supplementary Material.^36^^–^^39^

### 3.3 Endoluminal Application

**Fig. 5:**
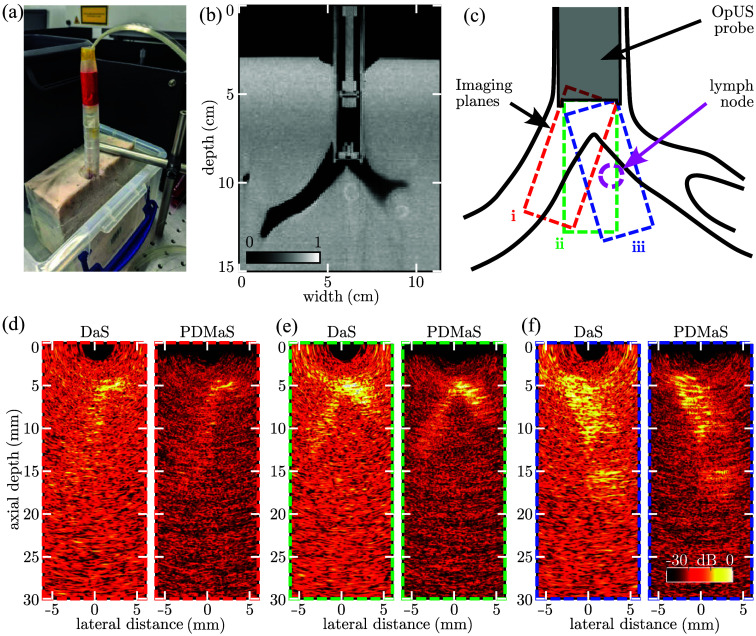
Endoluminal OpUS imaging of a tissue-mimicking endobronchial phantom. Photograph (a) and CBCT image (b) of the endoluminal-scale OpUS probe inserted into the tracheal lumen of a lung phantom. The OpUS probe is held in place by friction. (c) Schematic of the lung phantom indicating the lymph node inclusion and the approximate imaging planes i–iii shown in panels (d)–(f), respectively. (d)–(f) OpUS images acquired, at a frame rate of 10.1 Hz, from within the tracheal lumen of the lung phantom. Each image is displayed using both DaS and PDMaS image reconstruction. The lymph node inclusion is visible at a depth of ca. 12 mm in panel (f). All OpUS frames shown on the same logarithmic scale (Video 1, MP4, 17 MB [URL: https://doi.org/10.1117/1.JBO.31.1.016006.s1]).

The endoluminal-scale OpUS probe was inserted into the trachea of a tissue-mimicking, anatomically correct lung imaging phantom. CBCT imaging [Figs. 5(a) and 5(b)] was performed to confirm that the probe was positioned in front of the carina, and showed a spherical inclusion mimicking a lymph node located in front of the OpUS probe. The OpUS probe fully occluded the lumen, but the phantom was sufficiently pliant to allow for manual tilt and translation of the probe to alter the field of view [Fig. 5(c)]. As observed for previous freehand OpUS devices,^19^ the endoluminal-scale OpUS probe did not generate significant artifacts in the CT image, and exhibited a CT contrast (relative to air) indistinguishable from that of the tissue-mimicking material, which itself is a close match to *in vivo* human tissue.

OpUS imaging of the phantom under different orientations [Figs. 5(d)–5(f)] confirmed that the probe was capable of visualizing the branch point, lumen walls, and spherical inclusion mimicking the lymph node. The latter inclusion appears as a hypoechoic region and laterally offset from the branch point apex; this was due to a slight misalignment of the imaging plane relative to the phantom schematic of Fig. 5(c). The lumen walls were visualized up to a depth of ca. 20 mm, beyond which the lumen curved away from the imaging plane. As in Fig. 4, PDMaS beamforming consistently yielded higher imaging contrast than the DaS algorithm. Importantly, the OpUS images were acquired with the phantom fully filled with and submerged in water, confirming that the aHDE and airgap structure worked as intended even under full submersion. A video of the OpUS imaging of this lung phantom is provided in Video 1 (MP4, 17 MB).

## 4 Discussion and Conclusion

Incorporating an HDE into a freehand OpUS probe design enabled the fabrication of a probe comprising a higher channel count and denser source spacing while reducing the overall probe dimensions, resulting in the first endoluminal-scale OpUS imaging probe (diameter: 18.5 mm) capable of real-time, video-rate imaging at frame rates of up to 12 Hz. The probe comprised 144 sources (up from 64 in previously presented work^17^) at 62.5  μm source pitch (down from 400  μm) that satisfied the spatial Nyquist criterion for frequencies of up to 12 MHz. The corresponding absence of grating lobes and higher axial resolution resulted in a significantly improved image quality, and the high optical quality of the fabricated HDE resulted in greater uniformity across the fiber-optic ultrasound sources, broader bandwidths, and—despite a significantly larger surface area of the fiber-optic sources—comparable pressure levels as observed for a previously presented probe.^17^ This probe was readily fabricated in-house as the monolithic and uniform structure greatly facilitated HDE alignment and incorporation.

Imaging from within an anatomically correct lung ultrasound phantom confirmed the successful deployment of the probe in an endoluminal setting, as well as the reliability of the probe when operated fully submerged. The OpUS probe exhibited a CT contrast similar to that of soft tissue, which represents a further reduction in radiation attenuation compared with previous work,^19^ and due to the probe geometry and constituent materials, full compatibility with MRI imaging is also expected.

Due to a higher channel count and spatial source density, the endoluminal-scale OpUS probe avoided grating lobe artifacts, which enabled imaging of a tissue-mimicking phantom without averaging up to a depth of 25 mm [cf. Fig. 5 and Video 1 (MP4, 17 MB)], and consequently the system was still capable of video-rate imaging despite increased channel count and data volumes. The broader bandwidth of the emitted ultrasound and higher source density resulted in an improved axial resolution (ca. 125  μm). However, the smaller aperture of the probe (9.5 mm, down from 25 mm) resulted in reduced lateral resolution of ≥420  μm (up from 125  μm). Two different beamformers (DaS and PDMaS) were implemented in real time, and PDMaS consistently achieved higher image contrast but at reduced resolution.

Although the incorporation of an aHDE element resulted in superior image quality, a smaller probe form factor, and simplified probe assembly, the performance of the probe is limited by several factors. First, the use of a single fiber-optic ultrasound detector limits the image quality and its uniformity across the field of view, and placing this detector behind the photoacoustic generator membrane for mechanical protection introduced additional acoustical attenuation.^29^ However, incorporating multiple Fabry–Pérot-type detectors would require a full set of interrogation optics and digitizers per channel, which would result in a prohibitively bulky and costly experimental setup. Alternative fiber-optic sensor technology based on a fiber-coupled laser Doppler vibrometer^40^ could enable multidetector probes without the associated cost and experimental complexity and thereby significantly improve the image quality and uniformity. Second, the air gap used to prevent acoustic ringing measured 3 mm in thickness, resulting in eccentric OpUS sources measuring 2.3 mm in height. Decreasing this air gap to achieve an OpUS source height of 1.0 mm would retain the out-of-plane directivity yet decrease the source area and consequently increase the generated pressure levels—and hence the imaging depth. Third, the OpUS sources were positioned at a pitch of 62.5  μm by interleaving two sheets of fibers arranged cladding-to-cladding. A smaller pitch—and hence higher Nyquist frequency and ultimately axial resolution—could be achieved by extending this approach to more than two sheets or switching to fibers of smaller cladding diameter. The lateral resolution could be improved by increasing the source aperture, which could be nearly doubled without increasing the clam shell diameter. In addition, the optical fibers could be arranged aperiodically^18^^,^^41^ to further reduce grating lobes. However, each of these design alterations would complicate the assembly process and alignment requirements.

The use of a surface-HDE in this OpUS probe was motivated by their cost-effectiveness and range of available diffusion angles, and a mold-casting approach into a UV-curable adhesive was used to obtain a “negative” structure capable of withstanding the high intensity of the OpUS excitation light. This structure remained undamaged after hours of continuous imaging at full excitation power and hence proved highly reliable. However, the resulting structure was mechanically weak and required a backing substrate to prevent bending, and an additional protective layer to prevent water ingress and retain its holographic function in aqueous environments. In different applications, a flexible aHDE structure could prove useful and enable shaping of the excitation light in curved source apertures (e.g., a cylindrical ring transducer or a concave focused array). However, in the endoluminal-scale OpUS probe presented here, these substrates added additional propagation distance and corresponding beam divergence, as well as multiple reflection losses across the various optical surfaces. Alternatively, volume-HOEs could be used where the holographic structure is located within the element, which retain their holographic properties when wet and are hence better suited to aqueous environments, and could achieve higher transmission due to a reduced number of optical surfaces. In addition, HOEs offering other functionality have been presented that can, among others, collimate, focus, couple, or deflect light,^23^^–^^26^ and could further improve the source geometry in OpUS imaging. For instance, a custom HOE could achieve strong beam deflection over very short distances to achieve, e.g., a side-viewing OpUS probe without additional bulk—which would allow for endoluminal imaging of features within the lumen wall rather than within the lumen and expand the applicability of endoluminal OpUS imaging.

By incorporating an aHDE into a fiber-optic OpUS imaging array, a higher source density, smaller probe dimensions, and greater uniformity in acoustic performance across the aperture were achieved. The monolithic and thin structure of the HDE facilitated probe fabrication and miniaturization and enabled in-house assembly of an endoluminal-scale freehand OpUS imaging probe that was successfully demonstrated in an endoluminal setting. The OpUS probe exhibited superior image quality and greater application versatility yet retained the advantages (e.g., CT compatibility, flexible and handheld operation, real-time and video-rate imaging, high imaging resolution) established in previous work.^17^ Holographic optical elements, and diffuser elements in particular, hence significantly increase the clinical relevance of optical ultrasound imaging.

## Supplementary Material

10.1117/1.JBO.31.1.016006.s01

10.1117/1.JBO.31.1.016006.s1

## Data Availability

The code, data, and materials underlying the results presented in this paper are not publicly available at this time but may be obtained from the authors upon reasonable request.
