# Supplementary material for: Holographic diffusers for endoluminal-scale optical ultrasound imaging
Source: J Biomed Opt. 2026 Jan 23;31(1):016006. doi: 10.1117/1.JBO.31.1.016006 (PMC12827196; doi:10.1117/1.JBO.31.1.016006)
Supplement: Supplementary file 1 [file JBO_031_016006_SD001.pdf]

HOLOGRAPHIC DIFFUSERS FOR ENDOLUMINAL-SCALE OPTICAL ULTRASOUND IMAGING – SUPPLEMENTARY MATERIAL

1 aHDE FABRICATION

Figire 1 of the main manuscript demonstrated how a commercially available holographic diffuser element (HDE) was unable to withstand the high-intensity excitation light required for optical ultrasound excitation. To avoid the associated optical damage to the HDE, the holographic structure was imprinted into a UV curable adhesive, featuring a similar refractive index – and hence holographic performance – but significantly higher optical damage threshold. Supplementary Fig. S1 shows the steps performed to fabricate this adhesive-imprinted holographic diffuser element (aHDE), as well as further validation of its similar optical holographic performance.

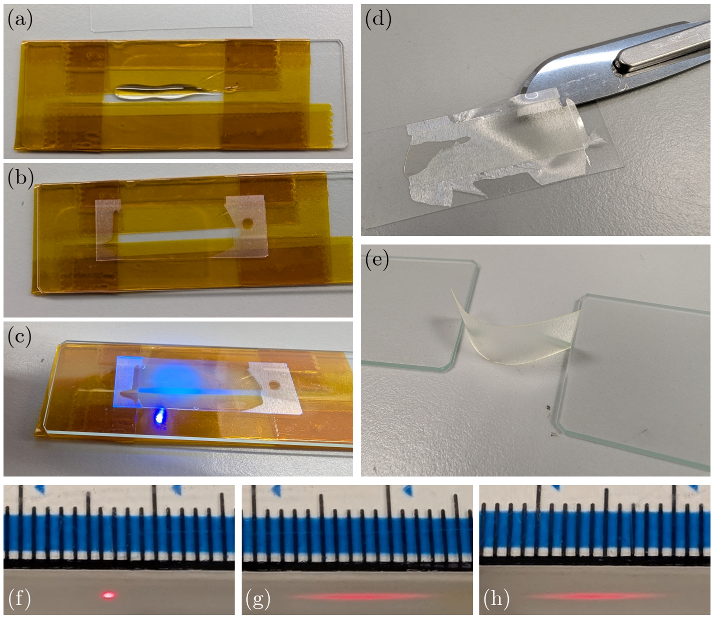

Figure S1. **Fabrication of the adhesive-imprinted holographic diffuser element (aHDE). (a)** A microscope slide was prepared by stacking two layers of polyimide tape (combined thickness: 104 µm) around a rectangular area acting as “reservoir”, and depositing the adhesive. **(b)** A section of the commercial HDE was prepared and placed over the adhesive reservoir, and pressure was applied to a second microscope slide placed on top of the commercial HDE to level and spread the adhesive across the reservoir area. **(c)** The adhesive was cured through UV illumination. **(d)** After peeling off the two microscope slides, a two-layer HDE-aHDE structure was obtained that was readily separated using a scalpel and manually peeled, leaving both the aHDE and commercial HDE functional and intact. **(e)** After trimming excess adhesive that was expulsed from the reservoir, a free-standing aHDE was obtained that is highly pliable, as demonstrated here by applying gentle pressure along its edges. **(f-h)** photographs of the light delivered by a laser pointer without HDE (f), or in the presence of the commercial (g) or adhesive-imprinted HDE (h) placed at a distance of 6.5 mm from the screen. Small and large ticks correspond to 1 mm and 5 mm increments, respectively.

2 ACOUSTICAL CHARACTERISATION

Figure 3(a) of the main text presented a compound image of the maximum intensity projection (MIP) across 144 field scans numerically propagated back to the transducer surface. However, as the source pitch (62.5 µm) was substantially smaller than the lateral extent of the sources (0.9 ± 0.3 mm), the spatial overlap between the MIPs for the 144 sources caused substantial blurring. Therefore, in Supplementary Fig. S2, these propagated field scans are separated across multiple panels to avoid this overlap.

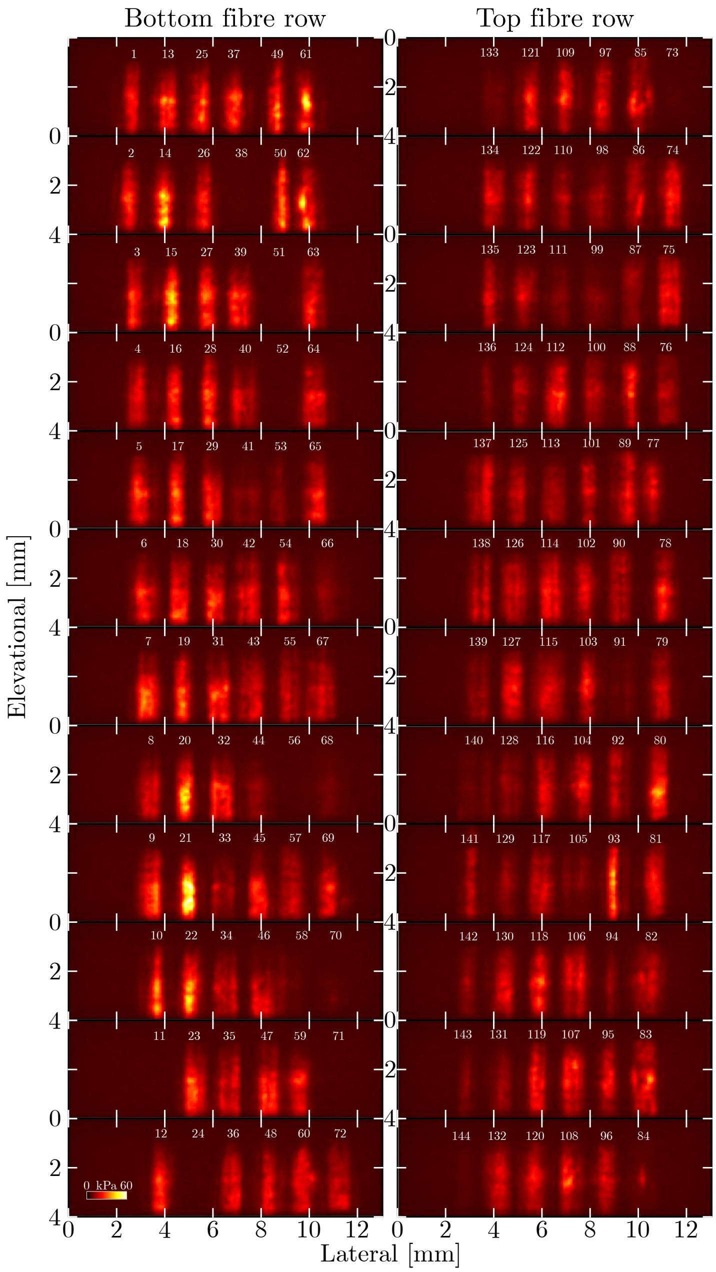

Figure S2. Compound maximum intensity projections of field scans numerically back-propagated to the transducer surface. Each panel shows six such field scans, and the fibre number corresponding to each scan is indicated above the source area. All panels shown using the same colour scale.

Variations in amplitude and occasional sources appearing absent (e.g. sources 38, 51, 52) are due to fluctuations in laser output power and hydrophone sensitivity; all 144 sources are accounted for and exhibit similar acoustical performance (*cf.* Fig. 3c-d of the main manuscript).

3 SIMULATED DATA

A simulation framework was developed to predict the performance of the OpUS imaging probe fabricated around an aHDE. This framework was based on the FOCUS toolbox [35,36] , which employs a numerically efficient closed-form expression of the spatio-temporal impulse response of round and rectangular piston transducers surrounded by a homogeneous medium that supports neither shear waves nor multiple scattering. The FOCUS toolbox does not rely on a near field approximation, and hence yields accurate near-field results at modest computational cost.

Pulse-echo B-scans were simulated by first generating a distribution of point scatterers, and subsequently computing the transient pressure generated by all discrete ultrasound sources in all point scatterer locations. For computational efficiency, the elliptical shape of the eccentric OpUS sources was approximated by a rectangular source with long and short axes matching those of the FWHM extent of the field scans propagated back to the transducer surface (*cf.* Fig. 3(b) of the main manuscript). The accuracy of this shape approximation has previously been shown to be accurate [9,40] . The resulting set of time traces are referred to as the "forward field". Band-pass filters were applied to match the simulations to acoustic measurements (*cf.* Fig. 3(d) of the main manuscript), and no additional noise was added.

Pulse-echo signals were then obtained by convolving the forward field with the spatio-temporal impulse response of the single, centrally-located fibre-optic detector. Given its small size, near-omnidirectional response and and near-uniform sensitivity [37] , this detector was modelled as an ideal point detector, and its impulse response hence reduced to the free space Green's function [38] . The pulse-echo signals originating from all point scatterers were coherently summed to yield a single pulse-echo A-line per source, and the set of A-lines were collected into a B-scan that was subsequently reconstructed into an image using the DaS algorithm.

To compare the expected performance gain of an OpUS imaging probe featuring an aHDE element to enable higher channel count and spatial source density, images were simulated for a grid of periodically spaced point scatterers (spacing: 2 mm lateral, 3 mm axial) using the geometries of the proposed endoluminal-scale probe and a previously presented freehand OpUS imaging probe [17] . As is observed in Supplementary Fig. S3, the higher channel count and spatial source density result in a significant reduction in grating lobe artefacts and increase in image contrast. However, the reduced aperture width yields a reduced lateral resolution.

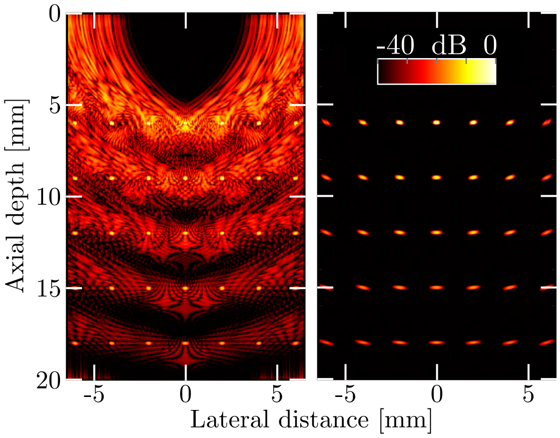

Figure S3. Simulated pulse-echo images for two OpUS probe designs. (left) A freehand OpUS probe [17] comprising 64 sources at a pitch of 400 µm. Each source measured 190 µm in width and 1.0 mm in height. **(right)** The endoluminal probe comprising 144 sources at a pitch of 62.5 textmu m. Each source measured 900 µm in width and 2.3 mm in height. Panels are shown on the same logarithmic scale.
